# CD177 Inhibits Neutrophil Extracellular Trap Formation and Protects against Acute Pancreatitis in Mice

**DOI:** 10.3390/jcm12072533

**Published:** 2023-03-27

**Authors:** Junxian Zhang, Xin Yang, Xingmeng Xu, Qinhao Shen, Fei Han, Qingtian Zhu, Keyan Wu, Aidong Gu, Dong Wu, Weiming Xiao

**Affiliations:** 1 Pancreatic Center, Department of Gastroenterology, Yangzhou Key Laboratory of Pancreatic Disease, Institute of Digestive Diseases, The Affiliated Hospital of Yangzhou University, Yangzhou University, Yangzhou 225000, China; 2 Operating Room of Anesthesia Surgery Center, West China Hospital, West China School of Nursing, Sichuan University, Chengdu 610041, China; 3 Department of Hepatobiliary Surgery, The Second Hospital of Nanjing, Nanjing University of Chinese Medicine, Nanjing 210008, China; 4 Department of Gastroenterology, State Key Laboratory of Complex Severe and Rare Diseases, Peking Union Medical College Hospital, Chinese Academy of Medical Science, Peking Union Medical College, Beijing 100730, China

**Keywords:** acute pancreatitis, neutrophil extracellular trap, reactive oxygen species, pathogenesis

## Abstract

The inflammatory immune response mediated by neutrophils is closely related to the progression of acute pancreatitis. Previous studies confirmed that CD177 is a neutrophil-specific marker involved in the pathogenesis of conditions such as systemic vasculitis, asthma, and polycythemia vera. Neutrophil extracellular trap (NET) formation is a specific death program by which neutrophils release nuclear DNA covered with histones, granule proteins, etc. It also plays an important role in host defense and various pathological reactions. However, the function of CD177 in regulating the generation of NETs and the development of acute pancreatitis (AP) is unclear. In our manuscript, CD177 was significantly elevated in blood neutrophils in patients and positively correlated with the AP disease severity. Then, recombinant human CD177 protein (rhCD177) could significantly improve pancreatic injury and the inflammatory response in AP mice, and reduce AP-related lung injury. Mechanistically, we found that rhCD177 could inhibit the formation of NETs by reducing reactive oxygen species (ROS) and myeloperoxidase (MPO)/citrullinated histone H3 (CitH3) release. For the first time, we discovered the potential of rhCD177 to protect AP in mice and inhibit the NET formation of AP. CD177 may be a potential treatment strategy for preventing or inhibiting the aggravation of AP.

## 1. Introduction

Acute pancreatitis is a common abdominal inflammatory disease that lacks specific clinical treatment [1,2]. Most clinical patients have mild and reversible cases of the disease, which is called mild acute pancreatitis (MAP) [3]. The acinar cell structure remains intact in MAP. However, in severe acute pancreatitis (SAP), injured acinar cells activate digestive enzymes and release factors that lead to the recruitment of inflammatory cells, along with the generation of multiple mediators, such as reactive oxygen species (ROS) and cytokines [4], resulting in systemic inflammatory response syndrome (SIRS) and even multiple-organ dysfunction (MODS) [5]. Therefore, the treatment of MAP patients is relatively conservative and requires only a short hospitalization, whereas that of SAP patients typically involves intensive care [6]. In recent years, SAP patients have incurred high health care costs, and the disease has shown a high mortality and great severity [7,8,9]. Therefore, new therapeutic targets through basic and clinical research necessitate additional investigation, which is probably crucial for delaying the disease progression and mitigating AP severity.

The underlying pathogenesis of AP presently remains unclear. Neutrophils are the most abundant leukocytes in human peripheral blood and represent the first line of defense against invading pathogens such as bacteria, fungi, and certain viruses [10]. The meshwork of chromatin fibers of NETs wraps around the bacteria and is studded with granule-derived antimicrobial peptides and enzymes, such as neutrophil elastase (NE) and myeloperoxidase (MPO). ROSs are also representative products of NETs. Peptidyl arginine deiminases 4 (PAD4) is also closely related to the formation of NETs and participates in the regulation of various inflammatory diseases. Previous studies have revealed that NETs induce trypsin activation and inflammation, promoting pancreatic duct obstruction and sometimes damaging pancreatic tissue [11]. Therefore, inhibiting neutrophil infiltration could ameliorate AP. Finding relevant targets for inhibiting NETs could promote novel treatments for AP and effectively reduce the risk of developing SAP.

CD177 encodes the neutrophil membrane glycoprotein (gp) NB1 [12], which belongs to the leukocyte antigen 6 (Ly-6) supergene family and is only expressed in neutrophils [13]. CD177 reportedly plays a key role in the genesis of several inflammatory diseases, such as arthritis [14], systemic lupus erythematosus (SLE) [13], pathogen-induced colitis [15], and inflammatory bowel disease (IBD) [16,17]; however, the function of CD177 in regulating NET formation and developing AP remains to be elucidated. For the first time, we collected AP patients to try to observe the correlation between CD177 and AP disease and clarified the modulatory role and mechanism of rhCD177 in AP mice through in vivo and in vitro experiments, which may be an important target for the clinical prevention and treatment of AP in the future.

## 2. Materials and Methods

### 2.1. Patients

All patient samples were obtained from the Department of Gastroenterology, Affiliated Hospital of Yangzhou University, from August 2020 to December 2021, which was interrupted by COVID-19. A total of 92 cases of AP patients were included in our studies. After excluding 42 patients by exclusion criteria (as shown in Supplementary Materials Figure S3), we divided AP patients into the AP without MODS (non-MODS AP) group (*n* = 29) and the AP with MODS (MODS AP) group (*n* = 21). All patients with acute pancreatitis were included in the study with the following inclusion and exclusion criteria. Exclusion criteria: (1) age <18 years or >75 years; (2) pregnancy AP; (3) diagnosis of tumor; (4) diabetes; (5) post-ECRP pancreatitis, etc. Patients with AP were diagnosed with two of the following three features (as per the revised Atlanta Classification, 2012). (1) Abdominal pain: clinically suggestive of acute pancreatitis; (2) serum lipase activity (or amylase activity) at least three times greater than the upper limit of normal; (3) radiological findings (USG/CT/MRI) suggestive of acute pancreatitis. AP patients with MODS (multiple organ dysfunction syndrome) were defined as failure of ≥2 organ systems (cardiovascular, pulmonary, and/or renal) persisting in a transient moment or more than 48 h by the improved Marshall scoring system.

### 2.2. Human Peripheral Blood Samples Processing

Patients’ venous blood was taken into blood collection tubes containing anticoagulants. Peripheral blood samples were mixed with the same amount of sterile PBS (PH: 7.4) and added with Lymphoprep (Alere Technologies AS, Oslo, Norway) sequentially to tubes (15 mL) for gradient centrifugation at 2500 rpm for 25 min. Cells that had aggregated in the lower layer were aspirated with a pipette. Neutrophils were obtained after lysing off the erythrocytes.

### 2.3. Mice

Approximately 20–25 g male C57BL/6J mice were purchased from the GemPharmatech Co. Ltd., Nanjing, China. Before experiments, the mice were housed in a specific pathogen-free room, with a controlled ambient temperature range of 25 ± 2 °C and a 12 h light/dark cycle, where they were fed standard rodent chow and water.

### 2.4. Ethics Statement

The Principles of Laboratory Animal Care (NIH publication No. 85Y50, revised 1996) were followed. All studies were approved by the Ethics Committee of Yangzhou University. (Approval no. 202202058).

### 2.5. AP Model Preparation and Sample Collection

Mice were divided into the normal control group (NC), caerulein (Cae)-induced AP group [18], and rhCD177-treated groups (Cae/rhCD177). All groups were intraperitoneally injected with caerulein (100 μg/kg) hourly for 7 h to induce the AP model, except for the control group. Recombinant human CD177 protein (rhCD177) was purchased from RD systems (Cat. No. 3505-CD-050, Purity >95%). To verify the protective effect of rhCD177 against AP, three concentrations of rhCD177 (3 ng, 0.3 ng, and 0.03 ng) were intraperitoneally injected 30 min before the first caerulein injection, forming the Cae/rhCD177 group. The remainder of the caerulein-induced AP group was simultaneously injected with an equal amount of PBS. Mice were sacrificed 12 h after the first caerulein injection and anesthetized with sodium pentobarbital (ip, 0.01 mL/g). Pancreatic tissues and pulmonary tissues were immediately harvested, and tissue samples were fixed with 4% paraformaldehyde for pathological and immunohistochemical staining (IHC). The remaining tissues were stored at −80 °C for immunofluorescence staining and Western blot detection. Blood was collected for amylase and lipase detection.

### 2.6. Histological Examination and Immunohistochemistry (IHC)

Pancreatic tissue samples were fixed for 48 h with 4% paraformaldehyde, then dehydrated, waxed, and baked at 60 °C for 30 min. The tissue sections were stained with hematoxylin eosin (H&E), following which gradient alcohol dehydration was conducted until xylene was transparent. Histological observation and photos were obtained under an upright fluorescence microscope (BX53, OLYMPUS, Tokyo, Japan). Histopathological scoring and analysis were performed by two independent pathologists via a blind method. According to the Schmidt method, pancreatic pathological injury score is a composite of three aspects: tissue edema, inflammatory cell infiltration, and acinar cell necrosis. The sample slides were dewaxed, dehydrated, antigen-recovered, and blocked by both endogenous peroxidase and normal goat serum. Slides were incubated overnight in a humid chamber at 4 °C with anti-myeloperoxidase antibody (1:50, Abcam, Cambridge, UK) and anti-F4/80 antibody (1:500, Abcam), following which they were incubated in biotinylated secondary antibody for 1 h. Finally, tissue samples were counterstained with hematoxylin. Images were acquired using a microscope (BX53, OLYMPUS, Tokyo, Japan). The positive staining areas were semi-quantitatively analyzed using Image J 1.x software.

### 2.7. Evaluation of Serum Enzymology and ELISA Determination

Cells were collected and centrifuged (1600 rpm, 4 °C, 5 min) to obtain the supernatant. The cell supernatant was pipetted into 96-well plates coated with antibodies for 12 h in advance. The levels of serum tumor necrosis factor alpha (TNF-α), and interleukine (IL)-6 were measured using a commercial kit according to the manufacturer’s instruction (Uncoated ELISA Kit, Invitrogen, Waltham, MA, USA).

Serum amylase and inflammatory factors were detected following the steps of manufacturer’s instruction book [19].

### 2.8. Flow Cytometry and ROS Determination

Flow cytometry slides were stained with the following antibodies: anti-CD45.2 (clone 104,1:200, Biolegend, San Diego, CA, USA), anti-CD11b (clone M1/701:200, Biolegend), anti-Ly6G (clone 1A8,1:200, Biolegend), anti-myeloperoxidase (clone EPR20257,1:500, Abcam), and MPO coupling Alexa Fluor488 (ab150077,1:1000, Abcam). The ROS content in the pancreatic tissue samples was detected using a DHE fluorescent probe (Sigma, St. Louis, MO, USA, D70N8, 1:2000). The cells were stained with DHE solution and incubated at 37 °C for 30 min. Cells were washed twice with PBS, then stained again with the above flow cytometry antibodies, and incubated at 4 °C for 30 min. Finally, the cells were collected using a Beckman DxFlex B5-R3-V3(Indianapolis, IN, United States) and analyzed using CytExpert2.4.0.28 for DxFlex.

### 2.9. Confocal Microscope

Mouse bone marrow neutrophils were extracted via gradient centrifugation. Cell climbing tablets were placed in the 12-well plates, after which the extracted cells were added. After being cultured in a cell incubator at 37 °C and 5% CO_2_ for 30 min, rhCD177 was added, and then Phorbol 12-myristate 13-acetate (PMA) was added 30 min later. After 4 h, the cells were fixed with 4% paraformaldehyde for 10 min and washed with PBS, and the cell membrane was broken using 0.25% TritonX-100. After another PBS wash, cells were inoculated on cell slides. The new slides were then incubated with PBS containing 5% goat serum for 1 h, and then with anti-histone H3 antibody (Abcam) overnight at 4 °C. One day later, slides were washed with PBS again and incubated at 25 °C for 2 h with Alexa Fluor 488 (green) labeled anti-rabbit IgG. Hoechst 33342 was used for nuclear staining after washing. Fluorescent immune samples were observed using a confocal microscope (Nikon A1R HD25, Tokyo, Japan) and analyzed using the NIS Elements AR 4.3 software.

### 2.10. Neutrophil Collection and Induction In Vitro

The marrow bone cells of C57/BL6J mice were extracted and then mixed with the cell diluent after lysing the red blood cells. The separation liquid was added to the 15 mL tube in advance, and the cell suspension was the slowly added. Centrifugation was conducted at 500–1000 g for 25 min at room temperature. After centrifugation, two ring-shaped milky white cell layers appeared in the centrifuge tube, where the upper layer was a mononuclear-cell layer and the lower layer of cells contained neutrophils. A pipette was used to carefully draw neutrophils into a clean 15 mL centrifuge tube, and the cells were washed with cell washing solution. Centrifugation was conducted at 250 g for 10 min. The cell supernatant was discarded, the above washing steps were repeated, and finally neutrophils were obtained. Freshly isolated bone marrow neutrophils were isolated from C57BL/6J male mice, then inoculated on 12-well plates (15 × 10^4^ cells/well) in RPMI 1640 medium, then cultured in a cell incubator at 37 °C and 5% CO_2_ for 30 min, and, finally, treated with 100 nM PMA or PBS for 4 h to construct an in vitro NETs model. After the incubation of the neutrophils with different doses of rhCD177 (0.5, 1, and 2 µg/mL) for 4 h, neutrophil infiltration and NET formation (MPO and ROS) were detected via flow cytometry.

### 2.11. Detection of Related Indexes of NETs In Vivo

The NETs content in pancreatic tissue was detected via immunofluorescence staining. The pancreatic tissues collected from experimental mice were fixed, embedded, and cut into 5 slices that were 1 μm thick. Pancreatic tissue sections were boiled in sodium citrate containing antigen repair buffer. Subsequently, the endogenous peroxidase and normal goat serum were blocked on the tissue. The tissue of the slide was dropped with anti-Myeloperoxidase (1:500, Abcam) and anti-histone H3 (1:500, Abcam) antibodies and incubated overnight at 4 °C with the primary antibody dilution of 1% goat serum. The slides were washed three times and incubated with the coupled secondary antibody 488 at 37 °C for 2 h. Finally, Hochest3342 was used to quench the sealing agent. After drying, the images were obtained using a confocal microscope.

### 2.12. Detection of Related Indexes of NETs In Vitro

Climbing tablets containing neutrophils were fixed with 4% paraformaldehyde and cultured with anti-histone H3 antibody solution for 12 h at 4 °C in the dark. The slide was then washed with PBS (pH = 7.4) and continuously shaken three times, each for 5 min. The slides were washed three times and incubated with the coupled secondary antibody at 37 °C for 2 h. Finally, Hochest3342 was used to quench the sealing agent, and, after drying, the images were obtained using a confocal microscope.

### 2.13. Western Blot Analysis

Pancreatic tissues or cells were extracted via ultrasonic crushing. The protein concentration was measured using a BCA protein kit (Thermo Fisher Scientific, Waltham, MA, USA) according to the manufacturer’s protocol. The proteins were subjected to 10% SDS-polyacrylamide gel electrophoresis (PAGE), and the proteins were transferred to a PVDF membrane, blocked with 5% skim milk, stored at room temperature for 2 h, and then incubated overnight at 4 °C with primary antibodies, such as anti-PAD4 (1:1000, Abcam) and anti-GAPDH (1:1000, CST). Membranes were washed with TBST thrice, each for 10 min. The next day, membranes were incubated with a secondary goat anti-mouse for 2 h at room temperature. Finally, membranes were washed thrice with TBST for 15 min each and protein bands were detected using the ECL Plus chemiluminescent system. Image intensity was analyzed using ImageJ 1.x software.

### 2.14. Statistical Analysis

The means of the two groups were compared using a Student’s *t*-test, whereas three or more groups of samples with normal distribution were compared using one-way ANOVA. Clinical data were analyzed by IBM SPSS 26.0 (Armonk, NY, USA). Statistical analyses were performed in the GraphPad Prism 8.0 software (GraphPad, San Diego, CA, USA), and the results are presented as the mean ± standard error (SE). The histogram data are presented as the mean ± standard error of the mean (SEM). *p* < 0.05 was considered statistically significant, denoted as * *p* < 0.05, ** *p* < 0.01, *** *p* < 0.001.

## 3. Results

### 3.1. CD177^+^ Neutrophils Increase in AP Patients Associated with Disease Severity

Previous clinical and laboratory data showed that AP complicated with MODS greatly increases the mortality of patients [20,21]. Therefore, we collected peripheral blood (PB) samples from a total of 92 AP patients. Based on the improved Marshall scoring system, we divided AP patients into the AP without MODS (non-MODS AP) group (N = 29) and the AP with MODS (MODS AP) group (N = 21). The clinical characteristics of the patients from different groups are shown in Table 1.

**Figure 1:**
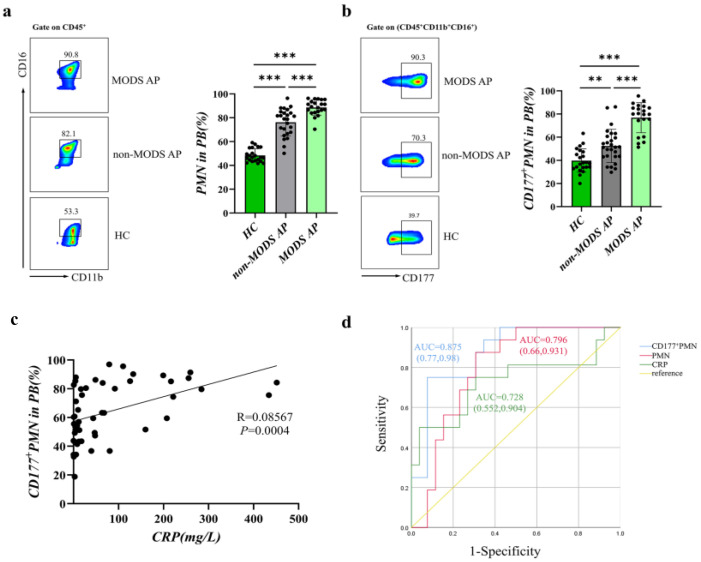
CD177^+^ neutrophils increase in AP patients associated with disease severity. The percentages of neutrophils (CD45^+^ CD11b^+^ CD16^+^) (**a**) and CD177^+^ neutrophils (CD45^+^ CD11b^+^ CD16^+^ CD177^+^) (**b**) in peripheral blood (PB) from AP patients and HCs were analyzed by flow cytometry. (**c**) CD177^+^ PMNs in AP patients showed a positive correlation with the levels of CRP. (**d**) ROC curve analysis of CD177^+^ PMNs, PMNs, and CRP levels for diagnosing MODS. Black dots represent samples. Statistical significance is indicated by ** *p* < 0.01, *** *p* < 0.001. AP, acute pancreatitis; PB, peripheral blood; PMNs, polymorphonuclear leukocytes; ROC, receiver operating characteristic; CRP, C-reactive protein; AUC, area under curve; MODS, multiple organ dysfunction syndrome.

As CD177 is a GPI-anchored glycoprotein normally expressed on a subpopulation of neutrophils [11], we analyzed CD177^+^ CD45^+^ CD16^+^ neutrophils from AP patients and healthy controls (HCs) (*n* = 22) by flow cytometry. As shown in Figure 1a,b, polymorphonuclear leukocytes (PMNs) and CD177^+^ PMNs are highly increased in AP patients compared with that of HCs. Moreover, the expression of CD177^+^ neutrophils was remarkably elevated in patients of the MODS AP group compared with that of the non-MODS AP group. It confirmed that CD177^+^ neutrophils are highly associated with the severity of AP. Furthermore, we found that the expression of CD177^+^ PMNs showed a positive correlation with C-reactive protein (CRP) levels (Figure 1c). Therefore, we performed ROC curve analyses using the presence or absence of MODS as the observed outcome. As the optimal cut-off value for CD177^+^ PMNs (%) was 72.81% (sensitivity 75.00%, specificity 92.30%), the area under the curve (AUC) was 0.875. We found that the CD177^+^ PMNs percentage was more accurate and sensitive in predicting progression to MODS compared to the PMNs percentage and CRP levels (Figure 1d, Table 2). In conclusion, CD177 could have promising clinical predictive value for AP development.

### 3.2. rhCD177 Successfully Inhibited NET Formation

**Figure 2:**
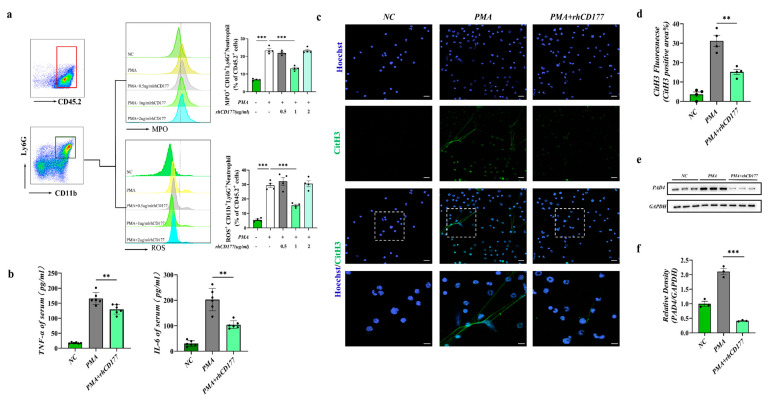
rhCD177 successfully inhibited NET formation. Bone marrow neutrophils isolated from six-to-eight-week-old C57BL/6 mice were cultured and stimulated with PMA (100 nM). (**a**) Representative flow cytometry plots and bar graphs depicting the proportion of neutrophils and the expression levels of MPO and ROS. Data are presented as means ± SEM (*n* = 4 per group, gating of CD45.2^+^ CD11b^+^ Ly6G^+^). (**b**) Supernatant levels of IL-6 and TNF-α were detected using ELISA (*n* = 6 each group). (**c**) Representative immunofluorescence image of CitH3 in magnification at 600× and 1000× (*n* = 4 per group). Scale bar = 20 μM. (**d**) Densitometric analysis of CitH3 fluorescence. (**e**) Western blot analysis of PAD4 levels in neutrophils. (**f**) Relative protein expression of PAD4; GAPDH was used as a control for protein loading. (*n* = 3 per group). Black dots represent samples. Statistical significance is indicated by ** *p* < 0.01, *** *p* < 0.001.

It was shown that the activation of neutrophils with the release of NETs can overactivate the immune system and thus cause MODS [22]. Subsequently, we used rhCD177 (purchased from RD systems, Cat. No. 3505-CD-050, purity >95%) to further explore the effect of rhCD177 on the NET formation in vitro. As shown in Figure 2a, the group treated with 1 µg/mL rhCD177 had the most conspicuous decrease in the amount of ROS and lowest MPO production than other groups. Hence, rhCD177 (1 µg/mL) was adopted to conduct functional and mechanistic research in the following in vitro experiments. Because the NET formation highly relies on ROS production by neutrophils, we confirmed that rhCD177 could suppress the neutrophil ROS release, thereby contributing to its inhibitory effect on NET formation. Similarly, the expression of inflammatory factors tumor necrosis factor alpha (TNF-alpha) and interleukin (IL)-6 in the cell supernatant of rhCD177 (1 µg/mL) group was drastically decreased compared with that of the control group (Figure 2b). Furthermore, NETs were stained with citrullinated histone H3 (CitH3) and Hoechst3342 in our study, as CitH3 is one of the key biomarkers of NETs. As shown in Figure 1c,d, PMA-treated neutrophils formed more NETs, whereas rhCD177 treatment significantly inhibited it. Peptidyl arginine deiminases 4 (PAD4), an enzyme family that has been proven to produce the most CitH3, was crucial for NET formation [23]. A consistent increase in the PAD4 expression level was detected in PMA-activated neutrophils; however, rhCD177 inhibited the increase (*p* < 0.05) (Figure 2e,f), consistent with the previous results.

### 3.3. Severity of AP Is Alleviated by rhCD177 in Mice

**Figure 3:**
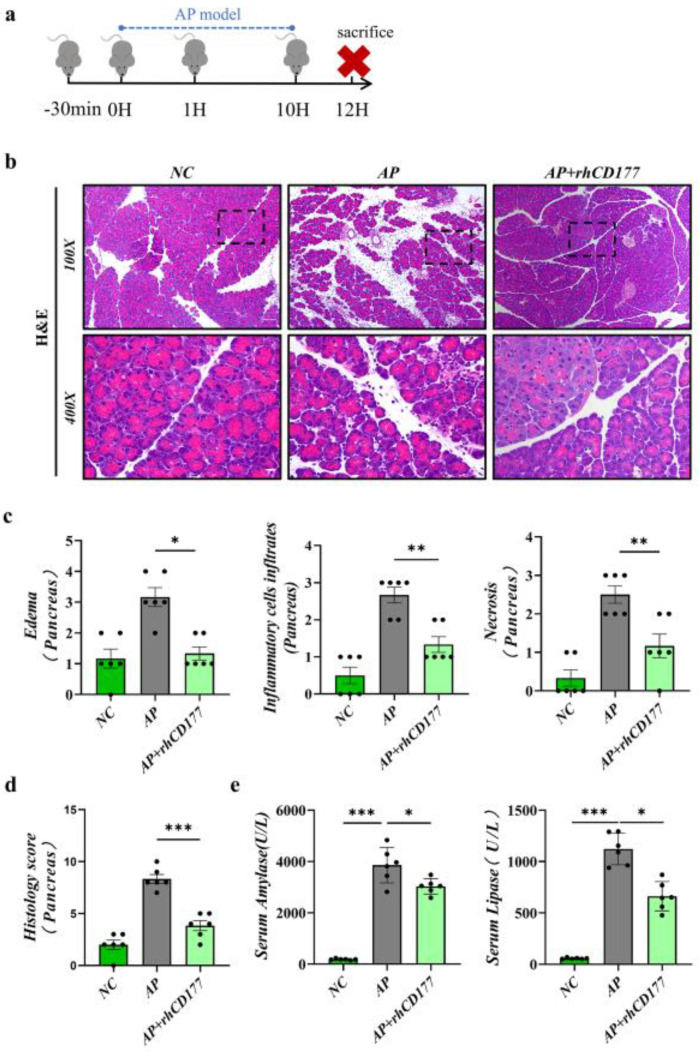
rhCD177 alleviates the severity of AP in mice (**a**) Flow chart of the establishment of Cae-indued AP model and rhCD177 administration in mice. (**b**) Representative HE stains of pancreatic tissues at magnifications 100× and 400×. Scale bar = 20 μM. (**c**,**d**) Pathological scores of pancreatic tissues. (**e**) Serum levels of amylase and lipase (*n* = 6 per group). Black dots represent samples. Data are presented as means ± SEM Statistical significance is denoted as * *p* < 0.05, ** *p* < 0.01, *** *p* < 0.001.

To determine the effect of rhCD177 on AP in mice, we injected rhCD177 into C57BL/6J mice by intraperitoneal injection (Figure 3a), and, after 30 min, mice were intraperitoneally administered caerulein (Cae) to establish AP models. Mice were sacrificed 12 h after the first dose of Cae. According to our results, evident increases in inflammatory cell infiltration, as well as edema and acinar cell necrosis, were observed in the AP group. Among previous dose gradient experiments, the medium-dose rhCD177 (rhCD177, 0.3 ng) exhibited the greatest protective effect, and the amylase and lipase levels in the serum were lower (*p* < 0.05), as shown in Figure 3b–d and Figure S1a–d. Based on these results, rhCD177 (0.3 ng) was used to conduct functional and mechanistic research in the following experiments.

### 3.4. Infiltration of Immune Cells Is Prevented in Pancreatic Tissues of AP Mice

**Figure 4:**
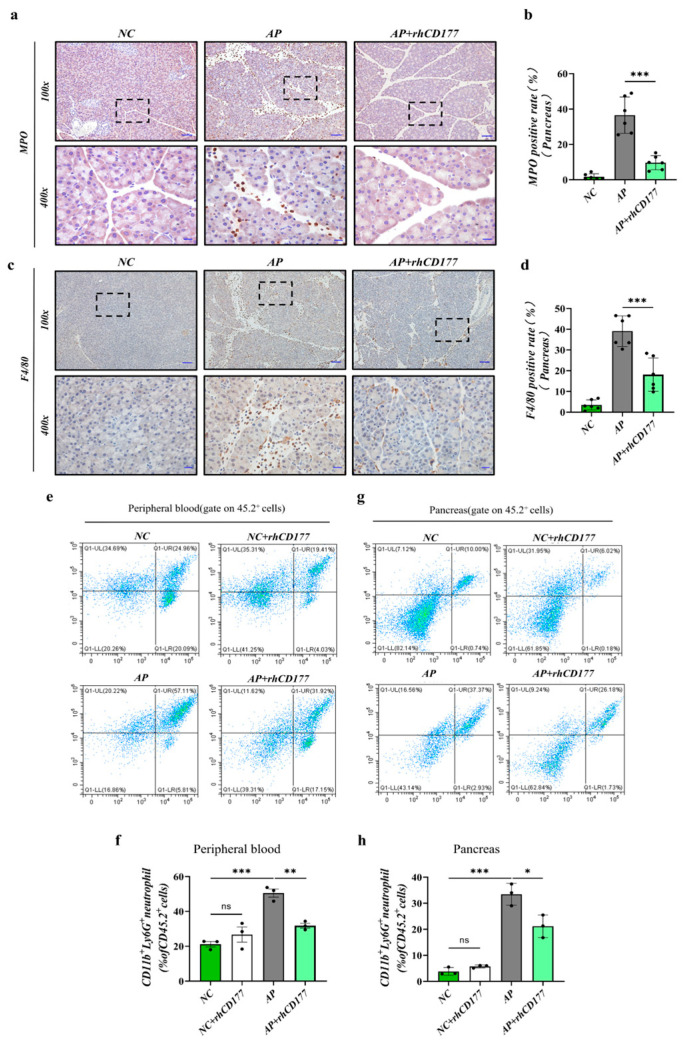
rhCD177 prevents the infiltration of immune cells in pancreatic tissues on AP mice. (**a**) Representative immunohistochemical images for MPO expression in the pancreas at magnifications 100× and 400×. Scale bar = 20 μM. (**b**) Quantification of MPO-positive pancreatic acinar cells in each group (*n* = 6 per group). (**c**) Representative immunohistochemical images for F4/80 expression in the pancreas at magnifications 100× and 400×. Scale bar =20 μM. (**d**) Quantification of F4/80 positive pancreatic acinar cells in each group (*n* = 6 per group). (**e**,**f**) Leukocytes from pancreatic tissue were isolated for staining and flow cytometry analysis. Representative flow cytometry gating of pancreatic neutrophils (CD45.2^+^CD11b^+^Ly6G^+^) is shown. (**g**,**h**) Percentages of CD11b^+^ly6G^+^ neutrophils from pancreas are shown as scatter plots. Black dots represent samples. Data are presented as mean ± SEM (*n* = 4 per group). Statistical significance is indicated by * *p* < 0.05, ** *p* < 0.01, *** *p* < 0.001.

We analyzed the expression of the neutrophil marker MPO and the macrophage marker F4/80 in the pancreas via IHC staining. As shown in Figure 4a–d, fewer MPO and F4/80-positive immune cells were observed with the addition of rhCD177 compared with those of the Cae group. Additionally, we isolated leukocytes from the pancreatic tissue of AP mice and observed that the number of activated neutrophils (CD45.2^+^ CD11b^+^ Ly6G^+^) was higher compared with that of the NC group. As shown in Figure 4e–h, the rhCD177 treatment significantly attenuated the infiltration of neutrophils in the pancreatic tissues and peripheral blood (*p* < 0.05). These results indicate that rhCD177 evidently decreased the amount of neutrophil infiltration and concentration of macrophages more than the AP group. This is consistent with our expectation that rhCD177 has a protective effect against AP.

### 3.5. rhCD177 Can Protect against AP-Induced Acute Lung Injury and Attenuate Inflammatory Responses

**Figure 5:**
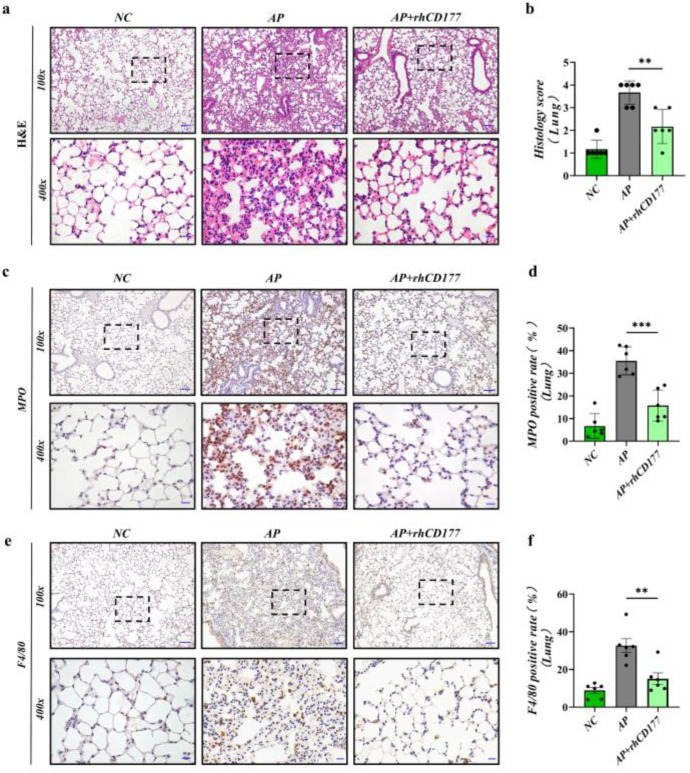
rhCD177 can protect against AP-induced acute lung injury and attenuate inflammatory responses. (**a**) Representative HE stains of lung tissues at magnifications 100× and 400×. Scale bar = 20 μM. (**b**) Pathological scores of lung tissues. (**c**) Representative immunohistochemical images for MPO expression in lung tissues at magnifications 100× and 400× Scale bar = 20 μM. (**d**) Quantification of MPO-positive cells in each group (*n* = 6 per group). (**e**) Representative immunohistochemical images for F4/80 expression in lung tissues at magnifications 100× and 400×. Scale bar = 20 μM. (**f**) Frequencies of F4/80 positive rate in lung tissues (*n* = 6 per group). Black dots represent samples. Statistical significance is indicated by ** *p* < 0.01, *** *p* < 0.001.

Acute lung injury (ALI) is the most common form of organ damage observed in AP. Approximately one-third of severe pancreatitis patients develop ALI or acute respiratory distress syndrome (ARDS) [24]; NETs play an important role in ALI [25,26]. Herein, we observed the pharmacological effects of rhCD177 on ALI in AP mice. First, the results of HE staining revealed disordered alveolar structures, thickened alveolar walls, obvious alveolar septums, interstitial edema, and infiltration in the lung tissues of mice in the AP group after rhCD177 administration compared with those in the NC group (Figure 5a,b). Fewer inflammatory cells were infiltrated, the alveolar septum was formed, and the capillary congestion status was significantly relieved. In addition, the numbers of MPO and F4/80-positive cells in AP mice were apparently increased, though rhCD177 could reverse this increase (Figure 5c–f).

### 3.6. rhCD177 Alleviates NET Formation in Pancreatic Tissues of AP Mice

**Figure 6:**
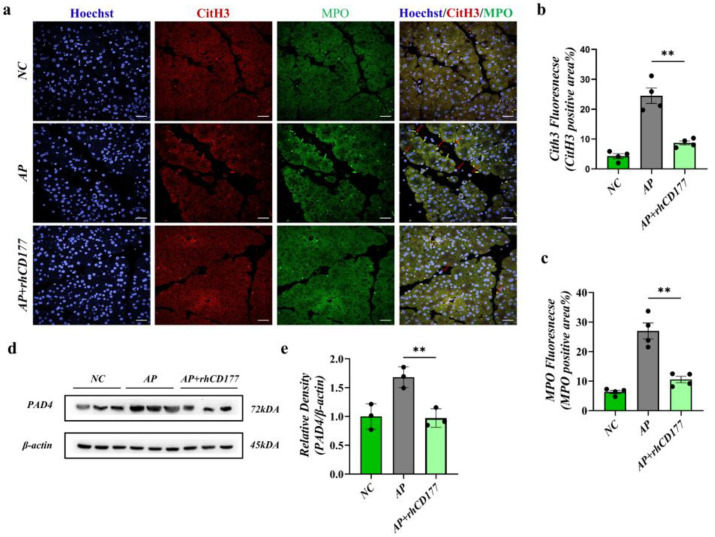
rhCD177 alleviates NET formation in pancreatic tissue of AP mice. (**a**) Representative immunofluorescence images of CitH3 and MPO at magnifications 600×. Scale bar = 20 μM. (**b**) Densitometric analysis of CitH3 fluorescence. (**c**) Densitometric analysis of MPO fluorescence (*n* = 4 per group). (**d**) Western blot analysis of PAD4 level in pancreatic tissue. (**e**) Relative protein expression of PAD4; β-actin was used as a control for protein loading (*n* = 3 per group). Black dots represent samples. Statistical significance is denoted as ** *p* < 0.01.

Considering the above results, we suggest that the expression of neutrophil infiltration (as shown by the IF staining of MPO) was significantly increased in AP mice compared to in the NC group (Figure 6a–c). Immunofluorescence staining revealed that CitH3 was released from infiltrating neutrophils. After rhCD177 treatment, the NET formation, represented by the co-localization of CitH3/MPO, was reduced. We then verified that the PAD4 expression was also decreased in pancreatic tissue after rhCD177 treatment using a WB assay (*p* > 0.05, Figure 6d,e). Overall, our results show that rhCD177 alleviated the cellular symptoms of AP by inhibiting neutrophil NET formation.

## 4. Discussion

**Figure 7:**
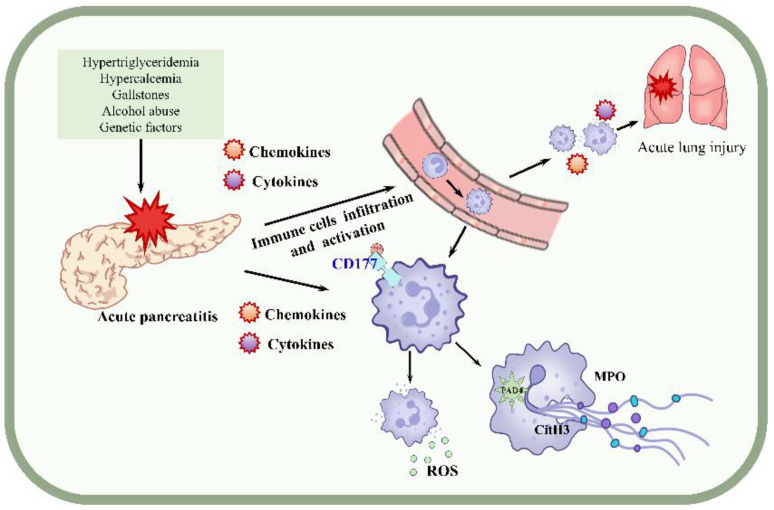
Mechanisms of CD177-regulated NETs in AP and AP-associated ALI.

We first found that the expression of CD177^+^ neutrophils is proportional to the severity of AP, and the application of rhCD177 could inhibit NET formation, thereby conferring protection against AP both in vivo and in vitro. Additionally, rhCD177 improved the symptoms of AP-induced ALI. Therefore, our study demonstrated that targeting the inhibition of NETs could be a promising approach for the clinical treatment of AP (Figure 7a).

As the primary defense system of the body, neutrophils play key roles in the occurrence and development of inflammatory responses in diseases. Studies have recently found that NET-mediated inflammatory responses can cause several inflammatory diseases. Moreover, neutrophils are principal phagocytes in the innate defense system, which ensnare and kill pathogens by releasing NETs. NETs are structures comprising chromatin and more than 20 kinds of primary and secondary granule proteins and enzymes, such as CitH3, MPO, and MMP-9 [27,28]. These granule proteins and enzymes are released into the extracellular space to kill pathogenic bacteria. Among them, the citrullination of histone H3 (CitH3) by PAD4 is a key signal for chromatin decondensation and NET formation. Moreover, CitH3 is considered as one of the most specific markers for NET formation assessment [29]. In addition, NETs depend on the production of ROS, which enables the release of neutrophil elastase and MPO from azurophilic granules. Hence, MPO is another reliable marker for the infiltration of neutrophils and appearance of NETs.

The inhibition of neutrophil activation and NETs is expected to ameliorate the symptoms of inflammation and prevent remote organ damage. Neutrophils participate in and aggravate the inflammatory response of AP through neutrophil NADP oxidase-induced oxidative stress and increase the activation of trypsinogen in PACs. In AP, neutrophils in peripheral blood migrate through the vascular endothelial cell space to the injured PACs, and the enhanced expression of ROS and NADPH oxidase further stimulates neutrophils to produce more pro-inflammatory cells [30,31]. However, many neutrophils lose the ability to clear the microbial infection, which can further aggravate the severity of damage at infection sites, thereby inducing a severe inflammatory response and even SIRS. In conclusion, the targeted inhibition of NETs may be a potential advancement in the treatment of AP.

CD177, which is only expressed by neutrophils, neutrophilic metamyelocytes, and myelocytes [32], has an important role in neutrophil transmigration through the endothelium owing to its high affinity for the adhesion molecule, platelet endothelial cell adhesion molecule-1 (PECAM-1) [33]. Studies have confirmed that CD177^+^ neutrophils represent a functionally activated population and protect against IBD through increased bactericidal activity and that CD177^−/−^ mice developed more severe colitis on DSS insults compared with wild-type mice [34,35]. These demonstrate that CD177 could be identified as a potentially useful surface marker of clinical outcome in inflammatory disease. In our studies, CD177^+^ neutrophils are strongly associated with the severity of acute pancreatitis. According to ROC curve analyses, the increased CD177^+^PMNs were proven to have diagnostic accuracy and be a currently promising predictor for the occurrence of MODS in AP. Based on these abovementioned studies, to further clarify the role of CD177 in AP, we established a mouse AP model by the intraperitoneal injection of Cae. Subsequently, we found that treatment with rhCD177 conferred protection against the caerulein-induced mice in AP, manifested by pathological changes, and attenuated amylase and lipase activities, as well as associated lung injury improvement. Consistent with the results of in vivo experiments, rhCD177 evidently inhibited NETs also in in vitro experiments. These recent findings suggest that rhCD177 has protective effects against AP. However, we did not explore the role of endogenous CD177 alterations in acute pancreatitis, which is a limitation of our study, due to the COVID-19 epidemic. There was not enough time to obtain knockout mice. In addition, we considered that rhCD177 had been commercialized and was relatively easy to obtain. Therefore, we applied rhCD177 for in vitro and in vivo studies. In consideration of safety issues, we also injected rhCD177 (0.3 ng) intraperitoneally into wild-type mice. We found that when the exogenous application of the rhCD177 (0.3 ng) is administered at safe concentrations, it could confer protective effects in the inflammatory response and tissue injury by decreasing the production of NETS, without affecting the development of various organs (pancreas, lung, heart, kidney, liver) in mice (as shown in Supplementary Materials Figure S2). Furthermore, neutrophils play a central role in SAP pathogenesis, and CD177 may be a remarkable predictor and potential target for the treatment of SAP.

In summary, our study confirmed that CD177 can predict AP with MODS, and that the exogenous supplementation of rhCD177 can improve inflammatory injury and prevent NET formation both in vitro and in vivo, providing several clinical intervention targets.

## Figures and Tables

**Table 1:** Demographic data and clinical characteristics of AP patients with or without MODS. Data are described as median (P25, P75) or *n* (%). MODS, multiple organ dysfunction syndrome; AAP, alcohol-induced AP; BAP, biliary AP; HLAP, hyperlipidemic AP; CTSI, computed tomography severity index; F, female; M, male; CRP, C-reactive protein. Others include idiopathic AP (IAP) and drug-induced acute pancreatitis (DIAP). * *p* < 0.05, ** *p* < 0.01, *** *p* < 0.001.

e	Non-MODS AP	MODS AP	*p* Value
e	29	21	
Sex (M/F)	15/14	15/6	0.165
Age (y, mean ± SEM)	47.00 (36.50, 57.50)	44.00 (32.75, 62.5)	0.316
Total leukocyte count (109/L)	9.89 (7.10, 12.76)	11.18 (9.42, 13.91)	0.047 *
Neutropill (%)	81.29 (67.22, 86.88)	88.87 (84.60, 92.01)	<0.001 ***
Lymphocyte count (109/L)	1.32 (0.90, 1.79)	0.96 (0.71, 1.11)	0.166
CRP (mg/L)	6.95 (2.95, 43.29)	76.94 (10.36, 212.10)	0.014 **
Etiology			
BAP	13	9	
HLAP	13	11	
AAP	3	0	
Others	0	1	
Length of hospitalization (d, mediam, 25–75%)	8.00 (5.00, 10.50)	11.50 (6.00, 18.00)	
CTSI	1.00 (1.00–2.00)	4.00 (2.25, 5.75)	
Improved Marshall scoring system	0.00 (0.00, 0.00)	2.00 (2.00, 2.75)	

**Table 2:** The AUC and the clinically optimal cutoff points of CD177^+^ PMNs, PMNs, and CRP for predicting the severity of AP.

Parameter	Cutoff	Sensitivity (%)	Specificity (%)	AUC
Non-MODS AP vs. MODS AP				
CD177^+^ PMN	72.810	75.000	92.300	0.875 (0.770, 0.980)
PMN	83.880	87.500	69.200	0.796 (0.660, 0.931)
CRP	89.060	50.000	96.200	0.728 (0.552, 0.904)

## Data Availability

The data that support the findings of this study are available from the corresponding author upon reasonable request (Weiming Xiao: wmxiao@yzu.edu.cn).

## Supplementary Materials

The following supporting information can be downloaded at: https://www.mdpi.com/article/10.3390/jcm12072533/s1. Figure S1: rhCD177 alleviates the severity of AP in mice in dose gradient experiments. Figure S2: rhCD177(0.3 ng) treatment had no effect on the main organs (pancreas, lung, heart, liver and kidney) of mice. Figure S3: The collection of cases of patients with AP. Figure S4: Difference in phenotype between PMA-treated neutrophils and untreated neutrophils.
