# Supplementary material for: CD177 Inhibits Neutrophil Extracellular Trap Formation and Protects against Acute Pancreatitis in Mice
Source: J Clin Med. 2023 Mar 27;12(7):2533. doi: 10.3390/jcm12072533 (PMC10094768; doi:10.3390/jcm12072533)
Supplement: Supplementary file 1 [file jcm-12-02533-s001.zip › jcm-2225501-supplementary.pdf]

Supplementary materials

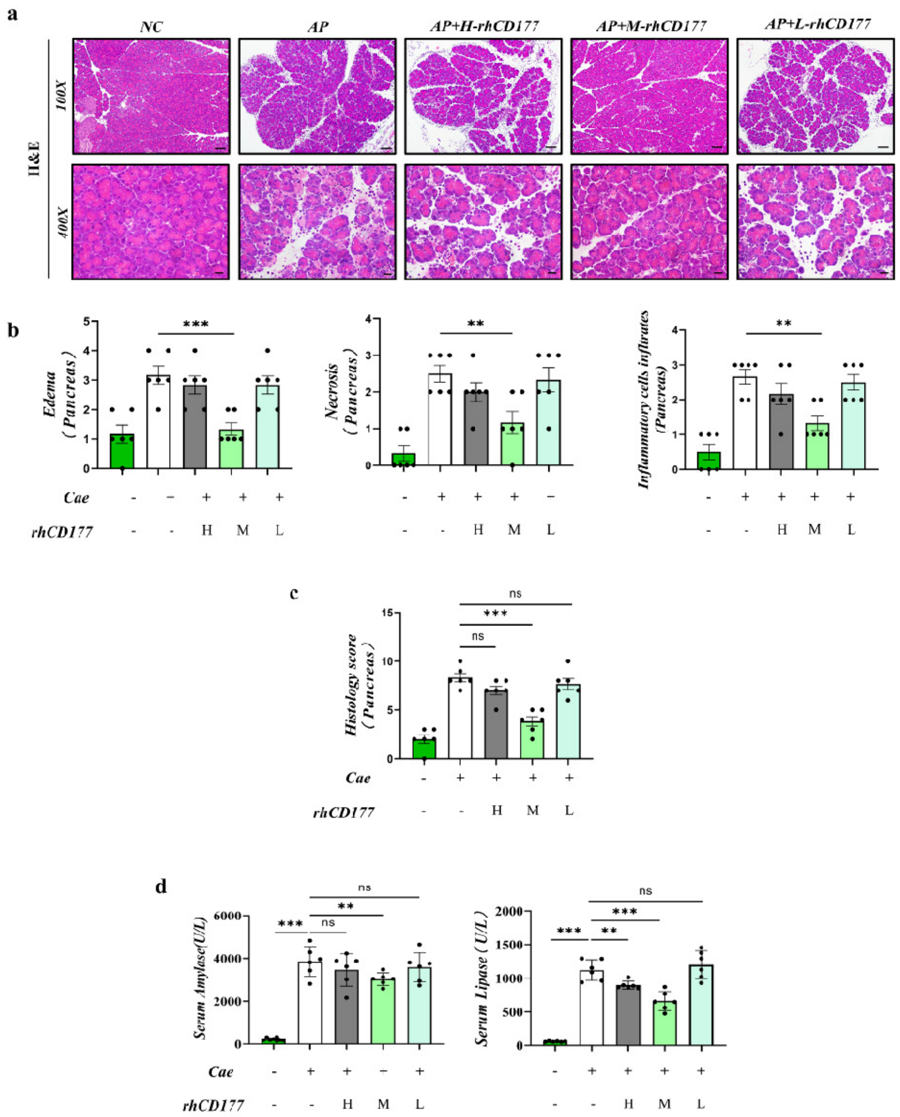

Figure S1. 1 rhCD177 alleviates the severity of AP in mice in dose gradient experiments. (a) Representative HE staining of pancreatic tissues at magnifications 100x and 400x. Scale Bar = 20μM. (b-c) The pathological scores of pancreatic tissues. (d) Serum levels of amylase and lipase. (N = 6 per group). Statistical significance is denoted as:, ***p* < 0.01, *** *p* < 0.001.

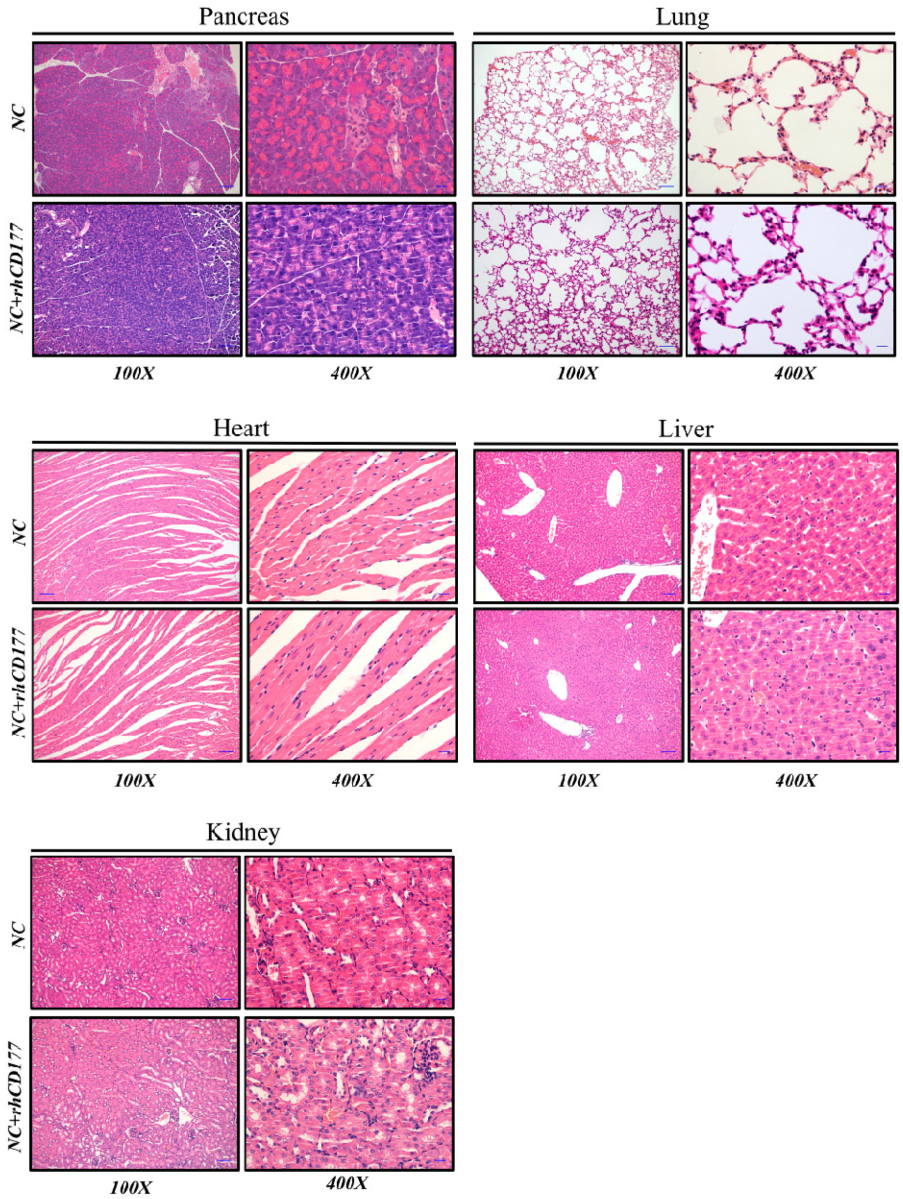

Figure S2. rhCD177(0.3ng) treatment had no effect on the main organs (pancreas, lung, heart, liver and kidney) of mice. Representative HE staining of pancreatic tissues at magnifications 100x and 400x. Scale Bar = 20μM.

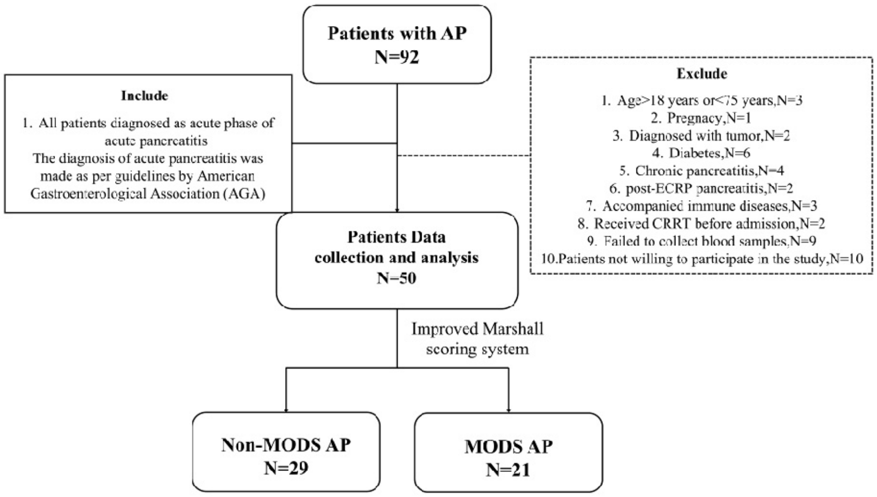

Figure S3. The collection of cases of patients with AP. Flow chart of the patients with acute pancreatitis (AP) in the clinical study.

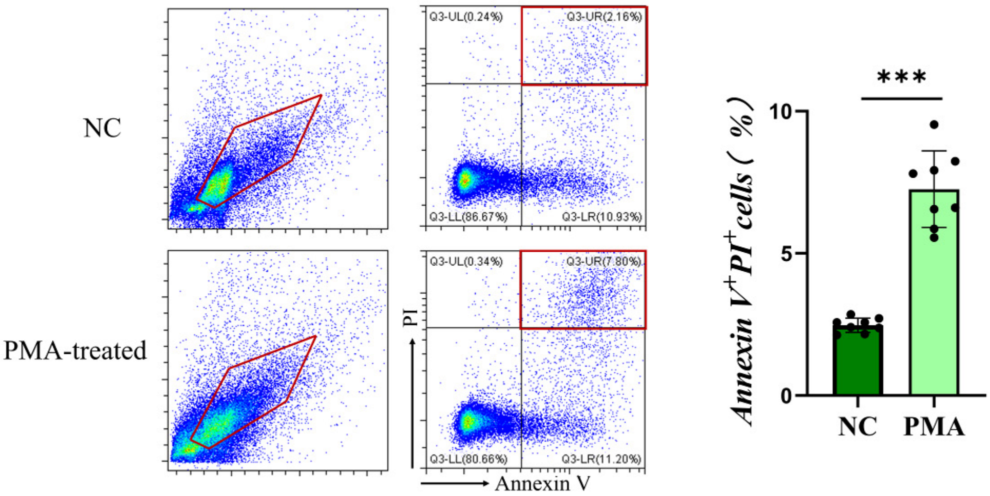

Figure S4. Difference in phenotype between PMA-treated neutrophils and untreated neutrophils. Flow cytometry with Annexin V / PI double staining was used to measure neutrophil apoptosis and necrosis. PMNs were exposed to 100 nMPMA for 4 h. The cells were made to react with FITC Annexin V and PI in the FITC Annexin V Apoptosis Detection Kit (BD) and were subsequently subjected to

flow cytometry. Both Annexin V-positive cells and PI-positive cells were increased in the PMA-treated neutrophils, compared with neutrophils of Normal Controls. Statistical significance is indicated by *** $p < 0.001$.
